# Sang-qi Granula Reduces Blood Pressure and Myocardial Fibrosis by Suppressing Inflammatory Responses Associated with the Peroxisome Proliferator-Activated Receptors and Nuclear Factor ****κ****B Protein in Spontaneously Hypertensive Rats

**DOI:** 10.1155/2013/721729

**Published:** 2013-09-22

**Authors:** Lan-Yu Chen, Chun-Shui Pan, Xiao-Hong Wei, Lin Li, Jing-Yan Han, Li Huang

**Affiliations:** ^1^China-Japan Freindship Hospital, China; ^2^Tasly Microcirculation Research Center, Peking University Health Science Center, Beijing, China

## Abstract

*Aim*. Sang-qi Granula (SQ) is a compound prepared from Chinese herbs and is currently used for treatment of hypertension in China. Given its protective effects on cardial function in decreasing blood pressure, we investigated the mechanism of protective effects of SQ on myocardium. *Methods*. 16 male normal Wistar-Kyoto rats and 16 spontaneous hypertension rats (SHR) were employed without medical treatment. 16 SHR were employed with SQ treatment. Rats in each group were sacrificed at two time points (8-week treatment and 16-week treatment). Blood pressure (BP), and heart weight/body weight (HW/BW) were measured. The expression of myeloperoxidase (MCP-1), ICAM-1, TNF-**α**, and CD68-positive cells was assessed. The interstitial collagen volume fraction (CVF), perivascular collagen volume area (PVCA), and the expression of TGF-**β**, Smad-3, PPAR**α**, **γ**, and NF-**κ**B (P65 and P50) were observed. *Results*. SQ significantly inhibited the elevation of the blood pressure and HW/BW of SHR. Next, SQ prevented myocardial fibrosis. Finally, a proinflammatory mediator associated with NF-**κ**B (TNF-**α**, ICAM-1, MCP-1, CD68), TGF-**β**, and Smad-3 related to collagen deposition, which is upregulated in SHR group, was significantly suppressed by SQ. Expression of NF-**κ**B was decreased in SHQ+SQ group compared to PPAR**α**, and **γ** expression was increased by SQ. *Conclusion*. Treatment with SQ ameliorates cardial fibrosis induced by hypertension by attenuating the upregulation of ICAM-1, TNF-**α**, MCP-1, TGF-**β**, Smad-3, P65, and P50 expression and improving PPAR**α** and PPAR**γ** expression level. The results suggest that SQ may be an option for preventing cardial fibrosis through PPAR signalling pathway.

## 1. Introduction

The World Health Statistics 2012 report indicated that the global average prevalence of hypertension is around 10%, and up to one-third of population in some Pacific Island countries is in hypertension. Even in Africa, however, more than 40% (and up to 50%) of adults in many countries are estimated to have high blood pressure. Long-term hypertension is an important and a prevalent contributor to morbidity and mortality from cardiovascular disease, and prolonged hypertension is accompanied by continuous vasoconstriction which can finally result in target organ damage, such as heart failure, stroke, and renal failure. 

Myocardial fibrosis is the result of chronic arterial hypertension and induces abnormality of cardiac function and arrhythmia. On the other hand, it is well accepted that vascular inflammation plays a major role in the cardiac fibrosis. Therefore, besides lowering blood pressure, attenuating of vascular inflammation is considered as an essential goal for the treatment of cardiac fibrosis following hypertension.

Sang-qi Granula (SQ) is a compound prepared from tradition Chinese herbs used for treating hypertension. The main pharmacological components of it are herba Taxillus chinensis, barbary wolfberry fruit, eucommia bark, cassiae torae, chrysanthemi indici, danshen root, kudzuvine root, and Alisma L. orientale Juzep. SQ has an anti-inflammatory action by alleviating the myocardial inflammation reaction in our previous study [1]. SQ's potent multiple functions and long history without adverse health effects and side effects make it a possible substitute for therapeutic treatment for myocardial fibrosis following hypertension.

The current project focuses on cardiac cell signaling related to transcription factors peroxisome proliferator-activated receptor (PPAR) and nuclear factor *κ*B (NF-*κ*B). PPARs belong to a superfamily of nuclear ligand-activated transcription factors that impact cell metabolism, cell differentiation, and inflammation. The nuclear hormone receptor superfamily consisting of 3 isoforms of PPAR*α*, PPAR*β*, and PPAR*γ*. PPAR*α* also exerts direct anti-inflammatory activity [2, 3]. Moreover, PPAR*α* is deactivated during cardiac hypertrophic growth [4] suggesting a role of PPAR*α* in regulating cardiac remodeling. In addition, systemic activation of PPAR*γ* by its agonist has been shown to prevent the progression of multiple cardiovascular diseases, such as hypertension, atherosclerosis, and chronic kidney disease by reducing inflammation and downregulating angiotensin II (AngII)-induced Ang II type 1 receptor (AT_1_R) expression [5–8]. Active NF-*κ*B promotes inflammation by promoting the transcription of various proinflammatory genes, including cell adhesion molecules, inflammatory cytokines, and chemokines. Cardiac NF-*κ*B activity is positively correlated with myocardial fibrosis [9, 10], and inhibition of NF-*κ*B activity limits myocardial fibrosis progression. PPAR and NF-*κ*B have been described as physiological antagonists: PPAR activation reduces NF-*κ*B/DNA binding [11]. In heart failure models, PPAR agonists reduce cardiac NF-*κ*B activity and reduce morbidity and mortality [12, 13].

In our study, SQ may alter cardiac PPAR and/or NF-*κ*B activity. If the SQ altered cardiac PPAR expression, it could also limit cardiac NF-*κ*B expression and associated cardiac inflammation and fibrosis. We then tested the hypothesis that SQ is also associated with increasing cardiac PPAR expression, decreasing NF-*κ*B expression, and reducing cardiac expression of cytokines and growth factors relevant to myocardial fibrosis pathogenesis.

## 2. Method

### 2.1. Animals

Thirty-two four-week-age male spontaneously hypertensive rats (SHR) were purchased from Beijing Vital River Animal Technique Limited Corporation (certificate no. SCXK 2006-0008) and sixteen four-week-age male Wistar-Kyoto (WKY) rats were obtained from SLAS Laboratory Animal (Shanghai, certificate no. SCXK 2007-0005). The animals were housed in cages at 22 ± 2°C and humidity of 40 ± 5% under a 12-hour light/dark cycle and received standard diet and water ad libitum. The experimental procedures were in accordance with the European commission guidelines (2010/63/EU). All animals were handled according to the guidelines of the Peking University Animal Research Committee. The protocols were approved by the Committee on the Ethics of Animal Experiments of the Health Science Center of Peking University (LA2011-38).

### 2.2. Animal Grouping and Medicine

SHRs were randomized into a SHR group (*n* = 16) and a SHR+SQ group (*n* = 16), with given 0.9% NaCl and Sang-Qi Granula (produced by China-Japan Friendship Hospital) at a dose of 100 mg·kg^−1^·d^−1^ treatment separately. WKY rats were fed with 0.9% NaCl and served as the control group (WKY group). The drug and 0.9% NaCl administration were performed via gastric gavage twice a day until the end of 16 weeks. 

### 2.3. Blood Pressure Measurement

Systolic blood pressure (BP) was monitored once every two weeks at 8 Am in a quiet room. After staying in a box at 29 ± 1°C for 10 min, the tail systolic blood pressure was measured using a blood pressure monitor (BP-98A, U0130163, Tokyo, Japan). Body weight was measured once a week.

### 2.4. Tissue Preparation for Histology

Half of the animals in each group (*n* = 6) were anesthetized with pentobarbital sodium (0.1 g/kg body weight) intraperitoneally at the 8th week. The rest of the animals were sacrificed at the 16th week. The hearts were rapidly excised and washed with saline on ice. The hearts were accurately weighed after the excess water on the surface was removed with filter paper. The ratio of the heart weight to body weight (HW/BW) was calculated. Then the left ventricular (LV) was divided into two parts: a section from the LV free wall was fixed in phosphate buffered 10% formalin overnight and embedded in paraffin for histopathological examination. The remaining part of LV was snap frozen in liquid nitrogen and stored at −80°C for subsequent protein. Blood samples were taken from abdominal aorta and the plasma was stored at −20°C until assay.

### 2.5. Masson Staining

The sections were stained with Masson and examined with a light microscope (BX512DP70, Olympus, Tokyo, Japan), according to the standard procedure [14]. Five fields in the ventricles of each animal were randomly selected, and the interstitial collagen volume fraction (CVF) and perivascular collagen volume area (PVCA) were quantified in the slides, in which the collagen fibers were visualized in blue.

### 2.6. Immunohistochemistry

The sections were incubated with antibody against CD68 after blocking with goat serum albumin. Incubation with PBS instead of the primary antibody served as a negative control. The samples were then incubated with horseradish peroxidase conjugated goat anti-rabbit immunoglobulin G (Zhongshan Goldenbridge Biotechnology Co., Ltd., China; dilution 1 : 3000). The images were captured by a digital camera connected to a microscope (BX512DP70, Olympus, Tokyo, Japan) and analyzed with Image-Pro Plus 5.0 software (IPP, Media Cybernetic, Bethesda, MD, USA). Five fields of left ventricle were examined for each animal. 

### 2.7. ELISA

At weeks of 8 and 16, animals from each group were anesthetized and the hearts were removed and homogenized in lysis buffer including protease inhibitor on ice. After being centrifuged at 20000 rcf for 60 minutes, the supernatant was collected for determination of MCP-1 content in heart tissues by ELISA, according to the manufacture's instruction. TNF-*α* and ICAM-1 content in rat plasma were evaluated by Elisa.

### 2.8. Western Blot Analysis

Western blot was performed as described previously [15]. Briefly, the heart was removed at weeks 8 and 16 and then was homogenized in lysis buffer including protease inhibitors. About 100 *μ*g of the supernatant was mixed with 4× sample buffer. The protein samples were separated on Tris-glycine SDS-PAGE in a reducing condition. The nuclear proteins were extracted using Nuclear and Cytoplasmic Extraction Reagents (NE-PER) kits (Thermo Scientific) according to the manual provided by the manufacturer. About 100 *μ*g of protein from each sample was separated by 12% SDS-PAGE. The primary antibodies used included those that directed against PPAR*α* (1 : 1000, Abcam, Cambridge, UK), PPAR*γ* (1 : 1000, Abcam, Cambridge, UK), NF-*κ*B P50 (1 : 1000, Abcam, Cambridge, UK), NF-*κ*B P65 (1 : 800, Cell Signaling Technology, Boston, MA, USA), ICAM-1 (1 : 200, Santa Cruz Biotechnology, Santa Cruz, USA), TGF-*β* (1 : 1000 Abcam, Cambridge, UK), Smad 3 (1 : 1000 Abcam, Cambridge, UK), GAPDH (1 : 2000, Cell Signaling Technology, Boston, MA, USA), and H3 (Histone3, 1 : 1000 Cell Signaling Technology, Boston, MA, USA). After washing with Tris-buffered saline containing 0.05% Tween-20, the membrane was incubated with horseradish peroxidase-conjugated secondary antibody (1 : 3000, Cell Signaling Technology, Boston, MA, USA) at room temperature for 60 min. The membranes were analyzed using the enhanced chemiluminescence system, according to the manufacturer's protocol and exposed in a dark box. The protein signal was quantized by scanning densitometry in the X-film by bioimage analysis system (Image-Proplus 5.0, Media Cybermetrics, Bethesda, MD, USA). 

### 2.9. Statistical Analysis

All parameters are expressed as means ± SD. Statistical analysis was performed using one-way ANOVA, followed by Turkey test for multiple comparisons. A probability of less than 0.05 was considered to be statistically significant.

## 3. Result

### 3.1. Effects of SQ on Systolic Blood Pressure (SBP) and Heart Weight/Body Weight (HW/BW) in SHR

**Figure 1:**
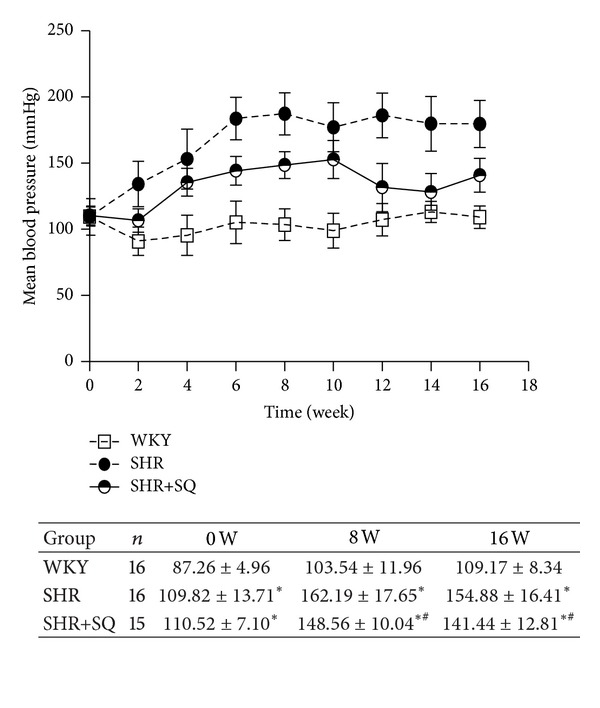
WKY: Wistar-Kyoto rats without treatments. SHR: spontaneous hypertensive rats without treatments. SHR+SQ: spontaneous hypertensive rats with Sang-qi Granula treatments. Data were expressed as mean ± SD of 12 animals.

**Figure 2:**
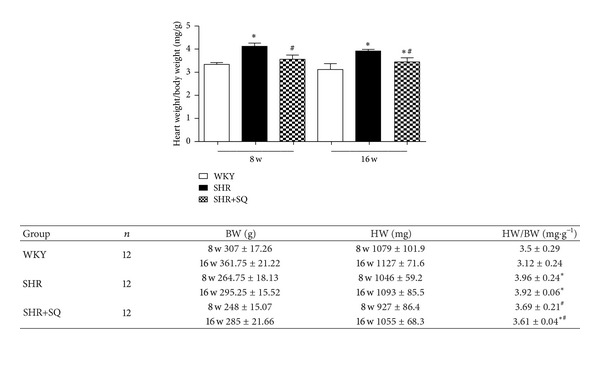
WKY: Wistar-Kyoto rats without treatment. SHR: spontaneous hypertensive rats without treatment. SHR+SQ: spontaneous hypertensive rats with Sang-qi Granula treatment. Data were expressed as mean ± SD of 12 animals. *P < 0.05 versus WKY  ^#^ *P* < 0.05 versus SHR.

Time-related changes in SBP for the three groups are shown in Figure 1. After 8 weeks of treatment, SBP in SHR group was significantly higher than that in WKY group and SHR+SQ group while SBP in WKY group was lower than that in SHR+SQ group. After 16 weeks, the trend is still the same, but SBP in SHR+SQ was much lower than before. We now observed the effect of SQ on HW/BW (Figure 2). The result showed that the ratios of HW/BW in SHR group were increased compared with WKY group and SHR+SQ group at weeks 8 and 16. In SHR+SQ group, HW/BW was a little higher than that in WKY group. But there was no difference between WKY and SHR+SQ group.

### 3.2. Effects of SQ on Interstitial and Perivascular Fibrosis in Left Ventricle of SHR

**Figure 3:**
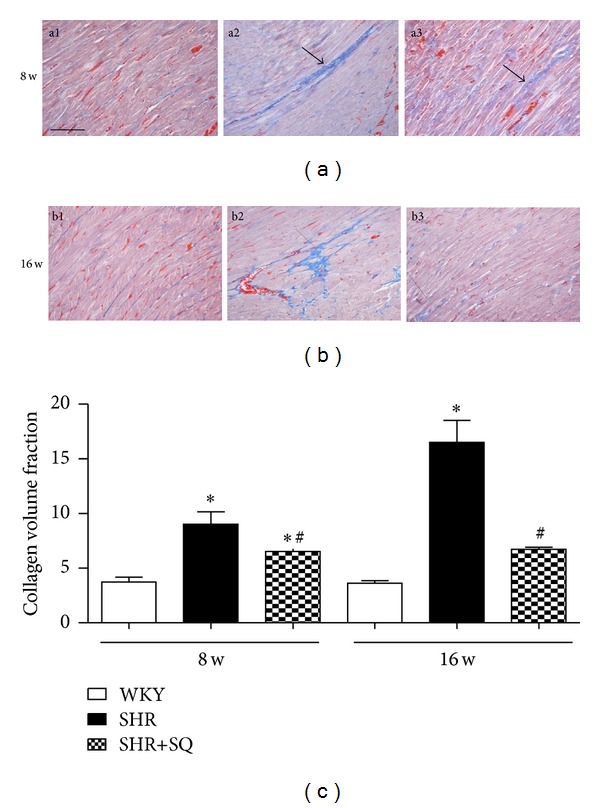
(a1) WKY: Wistar-Kyoto rats without treatment. (a2) SHR: spontaneous hypertensive rats without treatment. (a3) SHR+SQ: spontaneous hypertensive rats with Sang-qi Granula treatment. (b1) WKY: Wistar-Kyoto rats without treatment. (b2) SHR: spontaneous hypertensive rats without treatment. (b3) SHR+SQ: spontaneous hypertensive rats with Sang-qi Granula treatment. Data were expressed as mean ± SD of 3 animals. *P < 0.05 versus WKY ^#^ *P* < 0.05 versus SHR.

**Figure 4:**
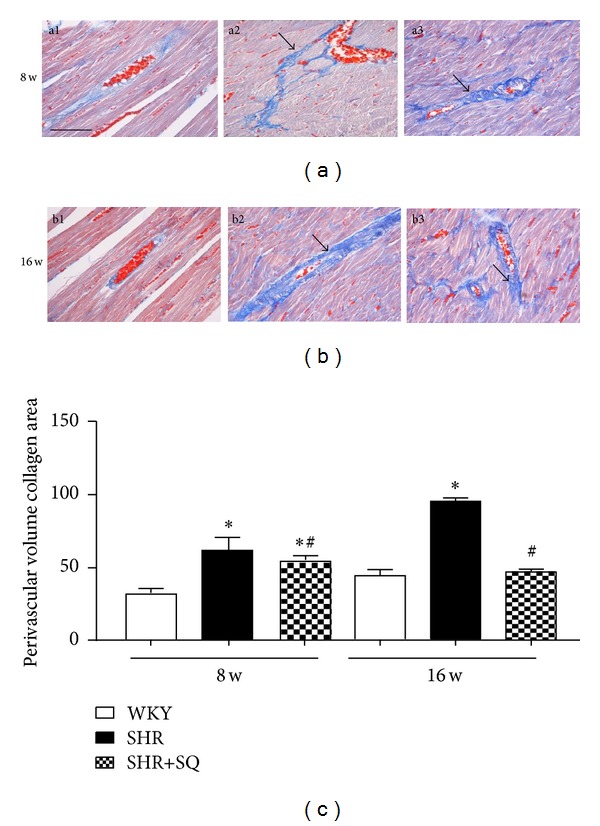
(a1) WKY: Wistar-Kyoto rats without treatment. (a2) SHR: spontaneous hypertensive rats without treatment. (a3) SHR+SQ: spontaneous hypertensive rats with Sang-qi Granula treatment. (b1) WKY: Wistar-Kyoto rats without treatment. (b2) SHR: spontaneous hypertensive rats without treatment. (b3) SHR+SQ: spontaneous hypertensive rats with Sang-qi Granula treatment. Data were expressed as mean ± SD of 3 animals. *P < 0.05 versus WKY ^#^ *P* < 0.05 versus SHR.

The SHR group showed a significant increase in the CVF compared to WKY group and SHR+SQ group (Figure 3). The collagen deposit immediately surrounding the vascular was also increased in SHR group compared to WKY group and SHR+SQ group (Figure 4). Treatment with SQ for 8 weeks and 16 weeks decreases both interstitial and perivascular collagen accumulation in SHR, and the effect of long-term treatment is more excellent. All of those factors indicated that SQ inhibited myocardial fibrosis by suppressing fibril deposition. 

### 3.3. SQ Increasing the Downregulated Expression of PPARs

**Figure 5:**
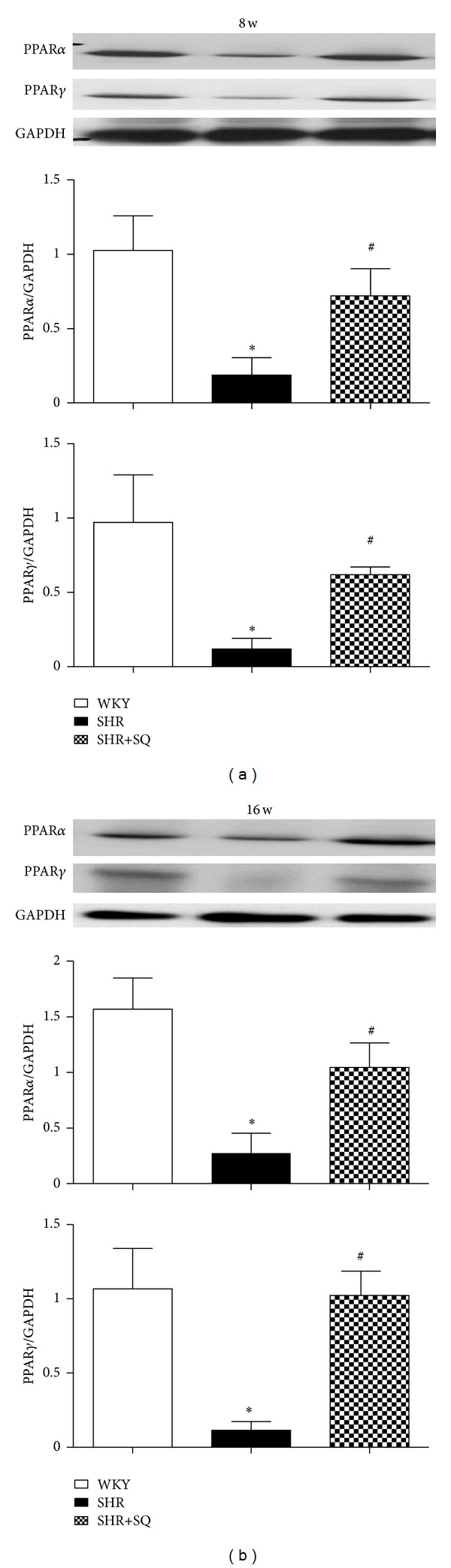
WKY: Wistar-Kyoto rats without treatment. SHR: spontaneous hypertensive rats without treatment. SHR+SQ: spontaneous hypertensive rats with Sang-qi Granula treatment. Data were expressed as mean ± SD of 4 animals. *P < 0.05 versus WKY ^#^ *P* < 0.05 versus SHR.

In SHR group, both PPAR*α* and PPAR*γ* expression were sharply decreased (*P* < 0.05) as compared with SHR+SQ group, which were statistically significant (Figures 5(a) and 5(b)). The expression level of PPAR*α* and PPAR*γ* in SHR+SQ group is similar to WKY group. The conserved upregulated expression in PPAR*α* and PPAR*γ* (relative to SHR group) could suggest a specific effect of SQ on PPAR*α* and PPAR*γ* expression.

### 3.4. SQ Reducing NF-*κ*B Expression

**Figure 6:**
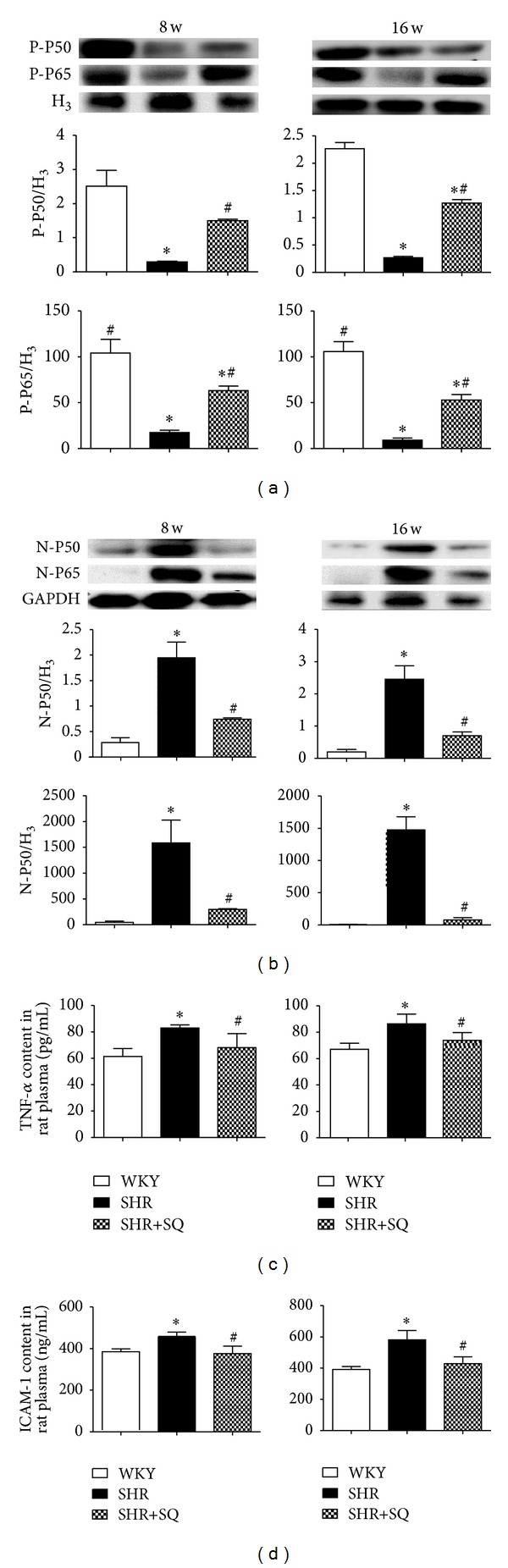
WKY: Wistar-Kyoto rats without treatment. SHR: spontaneous hypertensive rats without treatment. SHR+SQ: spontaneous hypertensive rats with Sang-qi Granula treatment. Data were expressed as mean ± SD of 4 animals. *P < 0.05 versus WKY ^#^ *P* < 0.05 versus SHR.

The protein levels of P65 and P50 proteins in the nucleus were significantly increased in SHR group but reduced in the SHR+SQ group. There was very weak expression of P50 and P65 in the WKY group (Figure 6). In contrast, the cytoplasmic levels of P65 and P50 in SHR group were significantly lower than those in WKY and SHR+SQ groups. In line with the alteration of the NF-*κ*B signaling, NF-*κ*B targeted cytokines, such as TNF-*α* and MCP-1 expression, were significantly elevated in SHR group.

### 3.5. SQ Attenuating Proinflammatory Mediators, Infiltration of Monocytes, and Inhibiting Collagen Deposition

**Figure 7:**
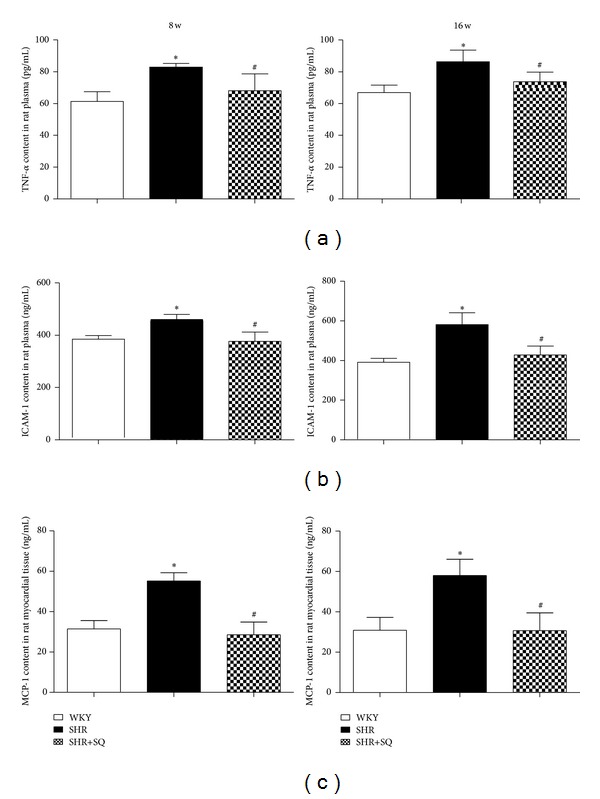
WKY: Wistar-Kyoto rats without treatment. SHR: spontaneous hypertensive rats without treatment. SHR+SQ: spontaneous hypertensive rats with Sang-qi Granula treatment. Data were expressed as mean ± SD of 7 animals. *P < 0.05 versus WKY ^#^ *P* < 0.05 versus SHR.

**Figure 8:**
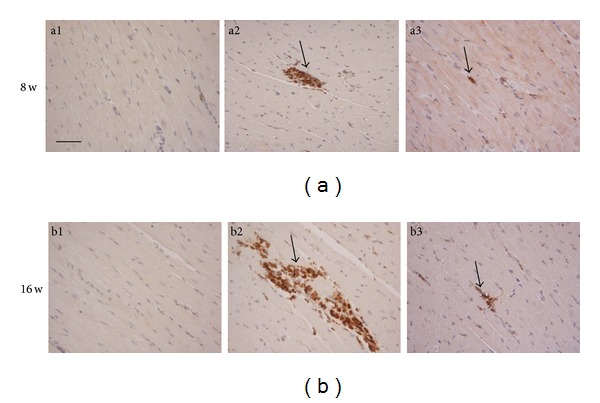
(a1) Wistar-Kyoto rats without treatment. (a2) spontaneous hypertensive rats without treatment. (a3) spontaneous hypertensive rats with Sang-qi Granula treatment. (b1) Wistar-Kyoto rats without treatment. (b2) spontaneous hypertensive rats without treatment. (b3) Spontaneous hypertensive rats with Sang-qi Granula treatment.

There was significantly increased expression of TNF-*α*, ICAM-1, and MCP-1 in SHR group whereas the upregulated expression was reduced in SHR+SQ group (Figures 7(a), 7(b), and 7(c)). TNF-*α* is a useful index of the level of cardiac inflammation and collagen. After 8 and 16 weeks of treatment with SQ, TNF-*α* in SHR+SQ group was less than that in SHR group, suggesting that SQ facilitated the degradation of collagen and decreased inflammation. Immunohistochemistry staining of CD68 is shown in Figure 8 to display monocyte infiltration and myocardial damage. The number of CD68 positive cells increased prominently in SHR group whereas only few CD68 positive cells exhibited in WKY group and SHR+SQ group. This result was in line with an increase in the expression of MCP-1 in SHR group.

### 3.6. SQ Inhibiting Collagen Deposition and Expression of TGF-*β*1 and Smad-3

**Figure 9:**
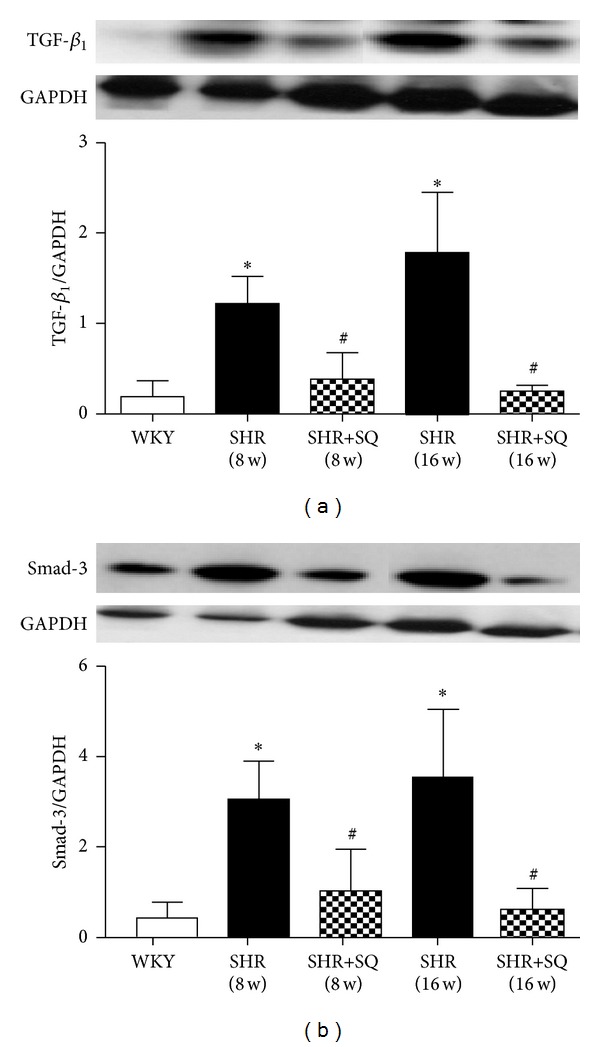
WKY: Wistar-Kyoto rats without treatment. SHR: spontaneous hypertensive rats without treatment. SHR+SQ: spontaneous hypertensive rats with Sang-qi Granula treatment. Data were expressed as mean ± SD of 4 animals. *P < 0.05 versus WKY ^#^ *P* < 0.05 versus SHR.

Results showed that, compared with SHR group, SQ had reduced TGF-*β*1 and Smad-3 expression, which were statistically significant. In contrast, the expression of TGF-*β*1 and Smad-3 in SHR group was sharply increased (Figures 9(a) and 9(b)). 

## 4. Discussion

Uncontrolled and prolonged elevation of BP pressure will lead to a variety of changes in the myocardial structure, coronary vasculature function, and the function of the cardiac conductive system. These changes in turn would induce the development of left ventricular hypertrophy (LVH), coronary artery disease (CAD), and systolic and diastolic dysfunction of the myocardium, as well as complications that manifest clinically as angina or myocardial infarction, cardiac arrhythmias (especially atrial fibrillation), and congestive heart failure (CHF). Although these diseases generally develop in response to chronically elevated BP, marked and acute elevation of BP can lead to accentuation of an underlying predisposition to any of the symptoms traditionally associated with chronic hypertension.

 Hypertension produces collagen deposition, changes referred to as myocardial fibrosis, which leads to depressed cardiac performance. Myocardial fibrosis is characterized by both quantitative and qualitative alterations of cardiac extracellular matrix (ECM) and hypertrophy of cardiocytes [16]. Cardiac fibroblasts phenotypically transformed myofibroblasts play a crucial role in the regulation of the ECM composition of the heart by synthesizing collagen and other matrix proteins [15, 17]. Myocardial fibrosis is a complex phenomenon reflecting the loss of the physiological reciprocal regulation between stimulatory (e.g., angiotensin II, endothelin I, catecholamines, aldosterone, basic fibroblast growth factor, insulin-like growth factor, etc.) and inhibitory factors (prostaglandins, nitric oxide, natriuretic peptides, etc.) acting on the turnover of fibrillar collagen [18]. In this study, the systolic blood pressure was decreased in SHR+SQ group compared with SHR group. The CVF and PVCA, important indexes of cardiac fibrosis, were obviously lower in SHR+SQ group. The results showed that the degree of fibrosis was significantly lower when treated with SQ.

 In several fibrotic processes, the role of inflammation has been clearly demonstrated. Several hypertension models revealed that perivascular fibrosis was often associated with inflammation cell around small arteries in the myocardium [19]. Profibrogenic cytokines are indeed released by inflammation cells [20]. Increased wall tension is involved in the extravasation of inflammatory cells around vessels, and then various cytokines from infiltrating cells, such as macrophages, become a trigger for perivascular and interstitial fibrosis [21]. Since Shahar [22] demonstrated that fibroblast proliferation in human interstitial lung disease was related to inflammatory cells, such as macrophages and lymphocytes, which can release cytokines that can act on cardiac resident interstitial fibroblasts.

It provides extensive pharmacological effect on cardiovascular system. It is verified that Sang-qi Granula plays as an important role in inhibiting ventricular hypertrophy in animal experiments in the past [5]. In traditional Chinese medicine (TCM), hypertension is classified as “dizziness”. Its basic pathogenesis is asthenia in origin and asthenia in superficiality. Asthenia in origin is the impairment of the liver and kidney. Asthenia in superficiality is the hyperactivity of liver-Yang, retention of phlegmatic dampness, and obstruction of collaterals by blood stasis. Consequently, we should apply, therapy strategies like nourishing the liver and kidney, calming the liver and suppressing Yang, eliminating dampness, resolving phlegm, and activating blood circulation to remove blood stasis. So herba taxilli, and eucommia bark were used to tonify the liver and kidney; barbary wolfberry fruit and Alisma L. orientale Juzep were used to nourish kidney Yin and clear deficient fire. Cassiae torae and chrysanthemi indici were used to clear liver heat and suppress liver Yang. *Salvia miltiorrhiza* root was used to nourish blood and promote blood circulation as well as communication between the heart and kidney. These herbs played an important role in harmonizing Yin and Yang, calming liver, suppressing endogenous wind, and promoting blood circulation. They can treat principal and subordinate symptoms simultaneously.

 However, as a therapeutic agent, the mechanism of SQ in preventing myocardial fibrosis still need to be investigated furtherly. The major process may be the inhibition of NF-*κ*B, a nuclear transcription factor that transactivates promoters of many inflammation infection and stress genes, including cytokines, and elicits a hypertrophic response in cardiac myocytes [23]. However, in resting cells, NF-*κ*B proteins are present in the cytoplasm as inactive heterodimers composed of two subunits, P50 and P65, and are bound to the inhibitory protein I*κ*Ba, which prevents it from translocating into the nucleus of the cell [24]. I*κ*Ba, the intrinsic inhibitor of NF-*κ*B, is phosphorylated and proteolytically degraded through a 26S proteasome. On stimulation, I*κ*Ba can facilitate NF-*κ*B translocation into the nucleus and regulates gene transcription [25]. NF-*κ*B translocates to the nucleus and binds to the I-kappa-B motif of the target gene, which causes activation of several factors involved in inflammatory responses. Various stimuli, including ischemia, free radicals, and cytokines, activate NF-*κ*B by inducing I*κ*Ba phosphorylation [11].

 NF-*κ*B also plays an important role in myocardial fibrosis. NF-*κ*B contributes to myocardial fibrosis pathogenesis because it regulates genes/proteins important for disease progression, including cytokines (e.g., TNF-*α*), interleukins (e.g., IL-6), growth factors (e.g., TGF-*β*), and adhesion molecules (e.g., intercellular adhesion molecule) [11]. It is postulated that after MI, activation of NF-*κ*B resulted in the expression of proinflammatory cytokines such as TNF-*α* and MCP-1 in cardiomyocytes, which promoted the infiltration of inflammatory cells, contributing to myocardial fibrosis [26]. TNF-*α* plays an important role in myocardial damage [27]. TNF-*α* stimulates the release of various inflammatory factors through autocrine and paracrine and induces cardiac myocyte apoptosis [28, 29]. Myocardial damage and apoptosis result in considerable infiltration of monocytes through MCP-1. MCP-1 is mainly released from apoptotic cells and recruits monocyte from the circulation to the apoptotic lesion. Infiltration of monocytes is a significant episode in the initiation of myocardial fibrosis, because the monocytes may differentiate into macrophages and participate in the healing process through production of growth factors, such as TGF-*β* and Smad-3 [30]. TGF-*β*/Smad 3 pathway plays an important role in cardiac remodeling [31]. In cardiovascular system, TGF-*β* is implicated in the development and progression of hypertension, heart failure, and other cardiovascular diseases [32, 33]. TGF-*β* is a cytokine with a broad range of regulatory effects on inflammation and cell proliferation, and it can regulate these processes through signaling pathway proteins called Smads [34]. In particular, the TGF-*β*/Smad 3 pathway can regulate inflammatory response. The pathway suppresses cytokine and chemokine expression in immune and endothelial cells and reduces macrophage chemotaxis [34]. In the process of ventricular remodeling, another aspect of the TGF-*β*/Smad 3 pathway is the regulation of fibroblast activity TGF-*β* induces phenotypic changes in fibroblasts to increase the expression of extracellular matrix protein [34]. As such, cardiac-specific deletion of NF-*κ*B activation inhibits inflammatory response which leads to a reduction in myocardial fibrosis via TGF-*β*/Smad 3 pathway. Given the critical role of NF-*κ*B signaling in cardiac fibrosis [27, 28], NF-*κ*B activation may represent an important mechanism for myocardial fibrosis.

 The present study demonstrated that SQ has direct beneficial effects on cardiac inflammation and collagen deposition of SHR. The protective effects are associated with decreasing infiltration of monocyte, NF-*κ*B, ICAM-1, TNF-*α*, MCP-1, and TGF-*β*1/Smads signaling molecules expression and increasing in PPAR*α* and PPAR*γ* expression. These findings indicated that the favorable cardiac effects of SQ on SHR are at least partly dependent on PPAR*α* and PPAR*γ* inhibiting inflammation through NF-*κ*B signaling. 

 Inhibiting of NF-*κ*B signaling may occur through different mechanisms and one of these mechanisms may be inhibiting the activation of PPAR*α* and PPAR*γ*. PPAR opposes NF-*κ*B activity in several ways. On one hand, PPAR improves I*κ*Ba transcription [29]. Delerive [35, 36] found that PPAR*γ* activators directed protein-protein-induced hepatic expression of I*κ*B, thereby preventing the P50 and P65 translocation into the nucleus. On the other hand, NF-*κ*B can inhibit PPAR binding to genomic PPAR response elements, thereby reducing PPAR transcriptional activity and the expression level of PPAR-related transcripts [37]. The PPAR receptors are expressed by multiple cell types in the cardiovascular system, including cardiac myocytes and fibroblasts.

Studies with PPAR*α* and PPAR*γ* confirm the inverse association of PPARs activity with NF-*κ*B activity. Diep also reported in human aortic smooth muscle cells that PPAR*α* activation inhibits cytokine-induced activation of a number of inflammatory genes, such as VCAM-1, COX-2, and IL-6 by negatively interfering with NF-*κ*B transcriptional activity [38]. Schiffrin demonstrated that activation of PPAR*α* resulted in inhibition of NF-*κ*B pathways that regulate expression of adhesion molecules ICAM-1 and VCAM-1 [39]. Recent studies have focused on ligands of PPAR*γ*, for its actions on the myocardium [40], and other studies have suggested a role for PPAR*γ* as an inhibitor of cardiac hypertrophy [36], that can decrease the NF-*κ*B binding activity. PPAR*γ* activators also directed protein-protein induced hepatic expression of I*κ*B, thereby preventing the P50 and P65 translocation into the nucleus [35, 36].

In conclusion, the protective effect of SQ on myocardium may partly account for a decrease of NF-*κ*B expression, inflammatory factors expression, myocardial damage, and MCP-1 expression which may diminish monocyte migration and infiltration and then inhibit cardiac fibrosis. 

## 5. Limitation

As a limitation of this study, it should be pointed out that another mechanism exists in myocardial fibrosis besides inflammatory responses through the NF-*κ*B signaling pathway. Furthermore, studies are necessary to determine the upstream and downstream pathways of NF-*κ*B and PPARs in order to elucidate the underlying molecular mechanism.

## 6. Conclusion

We demonstrated that prolonged hypertension-induced myocardial fibrosis was clearly prevented by treatment with SQ. From molecular analyses, we concluded that the reverse process of myocardial fibrosis was dependent on upregulation of PPAR*α* and PPAR*γ* expression, downregulation of NF-*κ*B expression, and suppressing TNF-*α*, ICAM-1, and MCP-1 production through the NF-*κ*B signaling pathway. However, as a therapeutic agent, the effect of SQ on restraining myocardial fibrosis still needs further investigation.
