# Correction to “Sang‐qi Granula Reduces Blood Pressure and Myocardial Fibrosis by Suppressing Inflammatory Responses Associated with the Peroxisome Proliferator‐Activated Receptors and Nuclear Factor κB Protein in Spontaneously Hypertensive Rats”

**DOI:** 10.1155/ecam/9830282

**Published:** 2026-01-05

L.‐Y. Chen, C.‐S. Pan, X.‐H. Wei, L. Li, J‐Y. Han, and L. Huang, “Sang‐qi Granula Reduces Blood Pressure and Myocardial Fibrosis by Suppressing Inflammatory Responses Associated with the Peroxisome Proliferator‐Activated Receptors and Nuclear Factor κB Protein in Spontaneously Hypertensive Rats,” *Evidence-Based Complementary and Alternative Medicine,* vol. 2013 (2013), https://doi.org/10.1155/2013/721729.

In the article titled “Sang‐qi Granula Reduces Blood Pressure and Myocardial Fibrosis by Suppressing Inflammatory Responses Associated with the Peroxisome Proliferator‐Activated Receptors and Nuclear Factor *κ*B Protein in Spontaneously Hypertensive Rats,” there was an error in Figure 4a related to the accidental duplication of the a2 panel. The error was introduced during the preparation of the article and should be corrected as follows:

Figure 4(a1) WKY: Wistar‐Kyoto rats without treatment. (a2) SHR: spontaneous hypertensive rats without treatment. (a3) SHR + SQ: spontaneous hypertensive rats with Sang‐qi Granula treatment. (b1) WKY: Wistar‐Kyoto rats without treatment. (b2) SHR: spontaneous hypertensive rats without treatment. (b3) SHR + SQ: spontaneous hypertensive rats with Sang‐qi Granula treatment. Data were expressed as mean ± SD of 3 animals. ^∗^ *p* < 0.05 versus WKY ^#^ *p* < 0.05 versus SHR.

**Figure:**
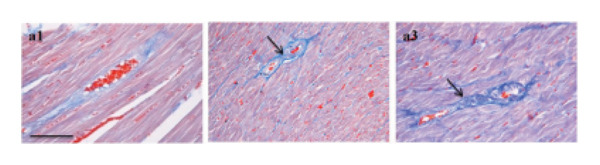
(a)

**Figure:**
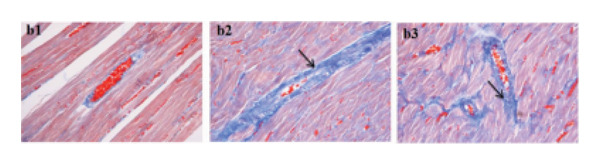
(b)

**Figure:**
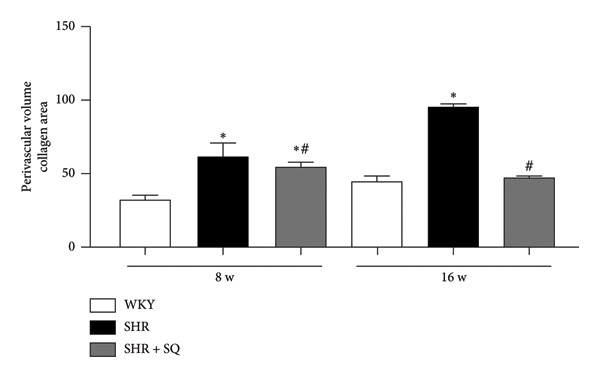
(c)

We apologize for this error.

